# A Clinical Audit of Cardiovascular Risk Factors and Disease in Patients with Rheumatoid Arthritis - SURF-RA

**DOI:** 10.31138/mjr.33.2.201

**Published:** 2022-06-30

**Authors:** Anne Grete Semb, Eirik Ikdahl, Anne M. Kerola, Grunde Wibetoe, Joseph Sexton, Cynthia S. Crowson, Piet van Riel, George Kitas, Ian Graham, Silvia Rollefstad, SURF-RA collaborators, Solbritt Rantapää-Dahlqvist, George Athanasios Karpouzas, Miguel A Gonzalez-Gay, Petros P Sfikakis, Maria G Tektonidou, Argyro Lazarini, Dimitrios Vassilopoulos, Bindee Kuriya, Carol Hitchon, Maria Simona Stoenoiu, Patrick Durez, Virginia Pascual-Ramos, Dionicio Angel Galarza-Delgado, Pompilio Faggiano, Durga Prasanna Misra, Andrew A Borg, Rong Mu, Erkin M Mirrakhimov, Diane Gheta, Karen Douglas, Vikas Agarwal, Svetlana Myasoedova, Lev Krougly, Tatiana Valentinovna Popkova, Alena Tuchyňová, Michal Tomcik, Michal Vrablik, Jiri Lastuvka, Pavel Horak, Helena Kaspar Medkova

**Affiliations:** 1 Preventive Cardio-Rheuma Clinic, Center for treatment of Rheumatic and Musculoskeletal Diseases [REMEDY], Diakonhjemmet Hospital, Oslo, Norway,; 2 Center for treatment of Rheumatic and Musculoskeletal Diseases [REMEDY], Diakonhjemmet Hospital, Oslo, Norway,; 3 Quantitative Health Sciences, Mayo Clinic Rochester, Rochester, Minnesota, United States of America,; 4 IQ Healthcare, Radboud University Nijmegen, Nijmegen, The Netherlands,; 5 Department of Rheumatology, Dudley Group of Hospitals NHS Trust, Dudley, United Kingdom,; 6 Cardiology, Trinity College Dublin, Dublin, Ireland

**Keywords:** cardiovascular disease, risk factor, rheumatoid arthritis, prevention

## Abstract

**Background and aims:**

Rheumatoid arthritis (RA) patients are at a high risk of atherosclerotic cardiovascular disease (ASCVD). This implies a need for meticulous CVD risk factor recording and control.

**Objectives:**

The aim was to evaluate the international prevalence of ASCVD in RA patients and to audit the prevalence and control of CVD risk factors.

**Methods:**

A **SU**rvey of cardiovascular disease **R**isk **F**actors in patients with **R**heumatoid **A**rthritis (SURF-RA) was performed at 53 centres in 19 countries in three continents between 2014 and 2019. CVD risk factors, medication, and physical and laboratory measurements were recorded. CVD risk was estimated using the ESC’s SCORE system.

**Results:**

Among 14503 RA patients in Western (n=8493) and Central and Eastern (n=923) Europe, Mexico (n=407), North America (n=4030) and Asia (n=650) (mean age 59.9 years, 74.5% female), ASCVD was present in 15%, varying from 2.5% in Mexico to 21% in Central and Eastern Europe. Sixty-two percent reported hypertension and 63% had a LDL-c of > 2.5 mmol/L. Mean BMI was 27.4 kg/m^2^ in the total cohort, highest in North America (29.7 kg/m^2^), and lowest in Asia (23.8 kg/m^2^). A sixth of patients were current smokers, and 13% had diabetes mellitus. Approximately 45% had an estimated high or very high risk of fatal CVD according to SCORE algorithm, and ¾ of patients had only ≤4/6 CVD risk factors at recommended target.

**Conclusion:**

Among RA patients across three continents, established CVD and CVD risk factors are common, although geographical variation exists. Furthermore, CVD risk factors often remain inadequately controlled.

## INTRODUCTION

The increased risk of cardiovascular disease (CVD) in patients with rheumatoid arthritis (RA) has been known for several decades and there has been a high focus on CVD prevention in this patient group. The elevated CVD risk in RA is only partly attributed to a high prevalence of traditional risk factors.^1^ RA is a chronic systemic inflammatory disease, and the chronic systemic inflammation is an independent CVD risk factor.^2,3^ CVD as a co-morbidity of RA is often overlooked and undertreated, and also management of CVD risk factors may be frequently neglected.^4,5^

Clinical audits can be applied to monitor data recording and management, measure clinical performance against guideline standards, and inform both appropriate treatments and the modification of recommendations to improve quality of care in routine practice.^6^ Robust data from audits can also be used by individual health professionals to improve their practice in response to information about their performance.^7^ Clinical audits are thus essential tools to monitor guideline implementation in clinical practice and to facilitate improved clinical performance.^8^ The **SU**rvey of cardiovascular disease **R**isk **F**actors (SURF) is a large and well-established audit of CVD risk factor management which has been performed in patients from primary care and in patients with coronary heart disease (CHD),^9,10^ and which currently is being performed in patients with stroke, chronic obstructive pulmonary disease, systemic lupus erythematosus and antiphospholipid syndrome.

The **SURF** in patients with **RA** (**SURF-RA**) is a new contribution to the SURF audit family in which CVD and its risk factors, RA treatment and use of CVD preventive medication are evaluated in RA patients. Since the risk of atherosclerotic CVD (ASCVD) and CVD death is increased in patients with RA, the overarching goal of SURF-RA is to improve CVD prevention in this vulnerable patient group.

## METHODS

Data from established cohorts and on consecutively examined patients in the time period 2014 to 2019 were included. The participating centres were divided into the following geographical areas: Western Europe, Central and Eastern Europe, Mexico, North America (USA and Canada), and Asia (**Supplementary Table S1**). The SURF-RA audit was performed to allow for quality improvement of CVD risk management in RA patients. It conforms to the ethical guidelines of the 1975 Declaration of Helsinki and was approved by the Data Protection Officer (DPO) at Oslo University Hospital (2017/7243) and a general data protection regulation (GDPR) evaluation was performed by the DPO at Diakonhjemmet Hospital (10/10-2018). Due to pseudonymization of data, a Data Protection Impact Assessment (DPIA) was deemed not necessary. Due to the quality assurance format, informed patient consent from the patient was not collected. Specifications on data protection, security and ethical aspects are described in **Supplementary Data 1**.

### Data collection

Data were collected on a one-page audit form which also included definitions of terms and instructions for clinicians. From the participating public and private outpatient clinics of rheumatology and cardiology, patients with RA aged >18 years were included. Apart from the age criterion, there were no exclusion criteria.

Demographic data included year of birth and sex. The following RA disease-related variables were recorded: Rheumatoid factor (RF) and anti–citrullinated protein antibody (ACPA) positivity, C-reactive protein (CRP), erythrocyte sedimentation rate (ESR), use of anti-rheumatic medication, as well as Disease activity score using 28 joints with either CRP or ESR (DAS28CRP and DAS28ESR).

The presence of objectively confirmed ASCVD was noted. Registered CVD risk factors were: smoking status (never/previous/current), physical activity (moderate meaning walking or equivalent 30 minutes 3–5 times/week, less or more than this), hypertension, hyperlipidaemia, obesity, diabetes mellitus, and the most recent CVD risk factor measurements. Lipids, glucose and glycated haemoglobin A1c (HbA1c) were recorded if there were measurements within 1 year of inclusion to the study. Moreover, use of lipid lowering agents and antihypertensive treatment was recorded.

CRP, ESR and lipid values were analysed according to each centre’s laboratory standards. For general CVD risk screening, fasting status has been shown not to influence the prognostic value of the blood sample^11^ and we therefore included both fasting and non-fasting lipid values.

The Cardiovascular Health Index Score (CHIS) was defined by control of six risk factors: blood pressure (BP) <140/90 mmHg or < 140/80 mmHg for patients with diabetes mellitus, low density lipoprotein cholesterol (LDL-c) <2.5 mmol/L, HbA1c <7% or, glucose <7 mmol/L if HbA1c was not available, non-/ex-smoker, body mass index (BMI) <25 kg/m^2^, moderate/vigorous physical activity. The number of risk factors at recommended targets was summed, ranging from 0 to 6. CHIS categories were defined as follows: poor if number of risk factors at recommended target/level was <3, intermediate: 3–4, good: 5–6.^10^

The presence and level of CVD risk factors were analysed separately for patients with and without established ASCVD (CHD, stroke, and/or peripheral arterial disease [PAD]).

### Risk of future CVD

To compare the risk of CVD across regions, we estimated the 10-year risk of a fatal CVD event by using the European Society of Cardiology’s Systematic Coronary Risk Evaluation (SCORE). Patients were then classified according to prevailing European Society of Cardiology Guidelines at the time of the data collection,^2^ into the following risk groups: Very high CVD risk: 1) Established CVD, and/or 2) estimated risk by SCORE >10%. High CVD risk group: 1) Presence of diabetes mellitus, and/or 2) TC >8.1 mmol/L, and/or 3) estimated risk by SCORE >5% and <10%, and/or 4) patients treated with lipid lowering medication. Moderate and low risk: estimated risk by SCORE of 1–4% and <1%, respectively.

### Statistics

The Kolmogorov-Smirnov test was used to evaluate the distribution of each parameter. Continuous variables are presented as mean with standard deviations (SD) or median with inter-quartile ranges (IQR) as appropriate. Categorical variables are presented as percentages. Between-groups comparisons of continuous variables were performed using the Kruskal-Wallis test, while the Chi-squared test was used for categorical outcomes. No imputation for missing data was done, and each variable was summarized using all reported data.

## RESULTS

### Patient demographics

The 14 503 patients included were from 53 centres across 19 countries in 3 world continents (**Supplementary Table S1**). The ethnicity of the participants in the various continent cohorts are also described in **Supplementary Table S2**. Since age, sex and disease duration were comparable across the different world regions and in the established a consecutively examined patient cohorts (**Supplementary Table S3a** and **S3b**), and time span for data collection was only 5.7 years, we merged the data from the established and consecutively examined patients into one cohort, which is used in the following evaluations. In the total cohort, the mean (SD) age was 59.8 (+13.6) years and there was a strong female preponderance (74.5%) (**Table 1**).

**Table 1.** Cardiovascular disease risk factors across world regions.

	**All**	**Western Europe**	**Central and Eastern Europe**	**Mexico**	**North America (USA and Canada)**	**Asia**	p-value*
Number of patients in region	14503	8493	923	407	4030	650	
Age mean(SD) [n]	59.8 (13.6) [14443]	60.7 (13.2) [8436]	58.8 (11.8) [923]	52.8 (11.6) [406]	59.4 (14.8) [4030]	55.7 (13.1) [648]	<0.001
Female sex %[n]	74.5 [14415]	74.1 [8412]	78.5 [917]	92.4 [407]	72.2 [4030]	77.3 [649]	<0.001
**Lipids** (mmol/L) mean (SD) [n]							
Total cholesterol	5.0 (1.1) [9359]	5.2 (1.1) [6082]	5.4 (1.2) [832]	4.6 (0.9) [406]	4.7 (1.1) [1510]	4.5 (1.0) [529]	<0.001
LDL cholesterol	2.9 (1.0) [9080]	3.0 (1.0) [5817]	3.2 (1.1) [699]	2.5 (0.7) [406]	2.5 (0.9) [1645]	2.7 (0.8) [513]	<0.001
HDL cholesterol	1.6 (0.5) [9093]	1.6 (0.5) [5835]	1.6 (0.5) [689]	1.4 (0.4) [406]	1.5 (0.5) [1650]	1.1 (0.3) [513]	<0.001
Triglycerides median (IQR)	1.2 (0.9–1.7) [9069]	1.2 (0.9–1.7) [5757]	1.3 (0.9–1.8) [728]	1.4 (1.1–1.8) [406]	1.3 (1–1.9) [1668]	1.1 (0.9–1.5) [510]	<0.001
LDL cholesterol >2.5 mmol/L (%) [n]	63.3 [9080]	67.9 [5817]	72.5 [699]	46.1 [406]	49.5 [1645]	56.3 [513]	<0.001
Total cholesterol >8.1 mmol/L (%) [n]	0.8 [9359]	0.7 [6082]	2.3 [832]	0.2 [406]	0.3 [1510]	0.6 [529]	<0.001
**Blood pressure** (mmHg) mean (SD)[n]							
Systolic blood pressure	127.9 (18.1) [10856]	128.8 (18.7) [5651]	132.2 (17.8) [923]	117.7 (17.5) [406]	126.6 (16.8) [3227]	127.1 (16.5) [649]	<0.001
Diastolic blood pressure	77.0 (12.2) [10791]	78.6 (12.8) [5587]	82.0 (10.9) [923]	74.3 (10) [406]	73.1 (10.8) [3227]	78.1 (9.4) [648]	<0.001
Blood pressure >140/90 mmHg (%) [n]	29.5 [10790]	31.9 [5586]	44.1 [923]	16.7 [406]	22.9 [3227]	28.7 [648]	<0.001
Blood pressure >140/90 and no use of anti-hypertensive medication (%) [n]	16.7 [8688]	18.3 [3493]	24.8 [921]	12.8 [406]	13.6 [3220]	15.1 [648]	<0.001
Hypertensive patients^a^ (%)[n]	62.3 [11838]	61.4 [6321]	75.3 [923]	36 [406]	66 [3540]	48.5 [648]	<0.001
**Diabetes** (%) [n]							
Diabetes type I and II combined	12.9 [11156]	12.3 [5418]	11.8 [905]	15.3 [405]	14.0 [3791]	10.7 [637]	0.017
Type I diabetes	0.7 [10606]	0.5 [4915]	1.1 [904]	0.0 [406]	1.0 [3744]	0.2 [637]	0.002
Type II diabetes	12.1 [10916]	12.2 [4928]	10.8 [915]	15.3 [405]	12.3 [4023]	10.4 [645]	0.387
**Glucose** (mmol/L) mean(SD)[n]	5.7 (1.8) [4833]	5.4 (1.4) [856]	5.5 (1.6) [858]	5.3 (1.6) [406]	6.1 (2.1) [2175]	5.1 (1.3) [538]	<0.001
Non-diabetics	5.3 (1.0) [3856]	5.2 (0.9) [744]	5.2 (0.9) [743]	5.0 (0.7) [342]	5.6 (1.1) [1560]	4.9 (1) [467]	<0.001
Diabetics	7.8 (3.3) [723]	7.1 (2.7) [96]	7.7 (3.2) [99]	6.9 (3.3) [62]	8.3 (3.4) [407]	6.6 (2.4) [59]	<0.001
**HbA1c** (%) mean (SD) [n]	5.8 (1.3) [2254]	5.7 (0.9) [881]	4.4 (1.5) [287]	5.8 (1.2) [166]	6.3 (1.4) [828]	6.0 (1.5) [92]	<0.001
Non-diabetics	5.3 (0.8) [1594]	5.5 (0.4) [772]	4.1 (1) [240]	5.5 (0.4) [137]	5.5 (0.6) [386]	5.3 (0.8) [59]	<0.001
Diabetics	7.1 (1.7) [595]	7.2 (1.8) [107]	6.3 (2.3) [40]	7.5 (2) [28]	7.1 (1.5) [388]	7.2 (1.7) [32]	<0.001
**Physical activity**[n]	3944	1769	920	404	328	523	<0.001
less than moderate %	42.5	44.7	24.5	70.0	64.6	31.9	
moderate %	41.1	38.3	52.4	20.3	20.7	59.7	
more than moderate %	16.4	17.0	23.2	9.7	14.6	8.4	
**Smoking** [n]	13172	7765	915	407	3443	642	<0.001
current %	16.5	19.2	28.5	8.1	9.7	7.8	
previous %	24.5	22.7	23.1	14.0	33.7	7.0	
never %	59.0	58.2	48.4	77.9	56.6	85.2	
**Physical measurements** mean (SD) [n]							
BMI (kg/m^2^)	27.4 (5.9) [11556]	26.6 (5.1) [7338]	27.7 (6.0) [854]	28.2 (5.7) [405]	29.7 (7.3) [2647]	23.8 (3.8) [312]	<0.001
Waist circumference (cm)	91.7 (15.4) [2686]	92.3 (16.6) [1132]	92.5 (15.5) [843]	92.2 (12.1) [260]	91.8 (12.7) [234]	84.1 (13.6) [217]	<0.001
**Premature CVD in family** % [n]	6.6 [4719]	6.2 [1937]	14.9 [922]	3.2 [407]	3.0 [803]	2.5 [650]	<0.001
**CVD risk categories (SCORE)**							
Number of patients [n]	6922	4179	676	402	1174	491 <0.001	
very high %	17.0	14.0	27.4	3.5	28.3	12.2	
high %	27.8	25.8	30.3	32.1	36.3	17.1	
moderate %	25.1	28.8	18.5	19.9	16.2	27.9	
low %	30.1	31.4	23.8	44.5	19.2	42.8	

**Abbreviations:**LDL, low density lipoprotein, HDL, high density lipoprotein, HbA1c, haemoglobin A1c, BMI, body mass index, CVD, cardiovascular disease, SCORE, systematic coronary risk evaluation, CV, cardiovascular, [n], number of patients with available data, SD, standard deviation, IQR, inter-quartile range,

* inter-regional differences,

a Hypertensive patients, BP >140/90 mmHg and/or use of a-HT.

### Rheumatoid arthritis disease specific variables

**Figure 1.**
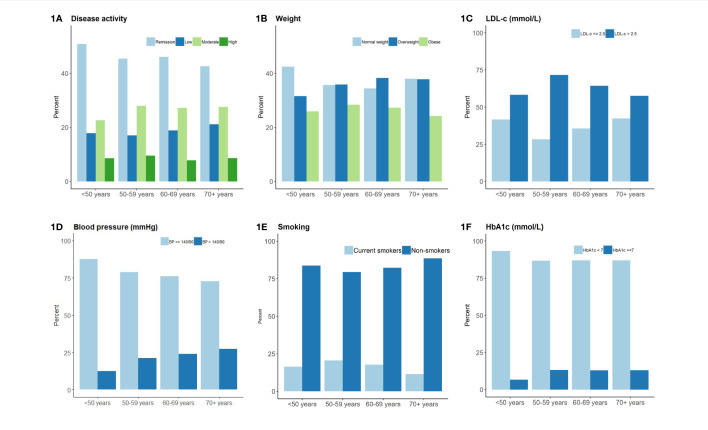
Disease activity and cardiovascular disease risk factors in rheumatoid arthritis patients across age categories. **(A)** Disease activity was divided into the following categories: remission when disease activity score with 28 joints including CRP (DAS28CRP) <2.6, low: DAS28 2.6–3.2, moderate: DAS28 3.3–5.1 and high: DAS28 >5.1. **(B)** Body mass index (BMI) was divided into the following categories; normal (18.5–24.9 kg/m^2^), overweight (25.0–29.9 kg/m^2^) and obese (>30.0 kg/m^2^). **(C)** Percentage of all patients having low density lipoprotein cholesterol (LDL-c) > or < 2.5 mmol/L. **(D)** Hypertension was defined as having blood pressure > 140/90 mmHg in both untreated and patients treated with antihypertensive medications. **(E)** Percentage of all patients being current or non-smokers. **(F)** Percentage of all patients having haemoglobin A1C (HbA1c) ≥ or < 7 %.

More than half of the patients were RF and/or ACPA positive. Disease duration was quite similar across the geographic regions, the mean varying 9.9 - 12.6 years (**Supplementary Table S2**). RA disease activity evaluated by composite measures was on average low, and this was stable with increasing age (**Figure 1A**). Inflammatory markers and DAS28-ESR were highest in Asian RA patients (**Supplementary Table S2**).

### Cardiovascular disease

**Figure 2.**
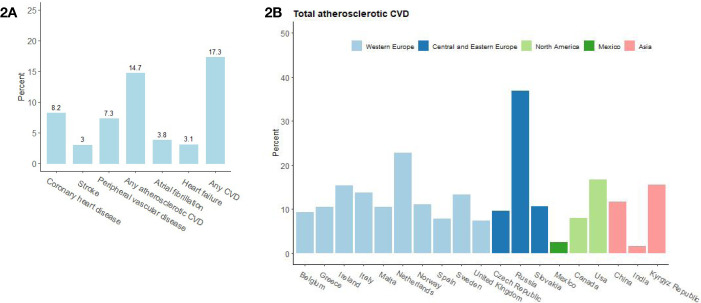
Cardiovascular disease in patients with rheumatoid arthritis (RA). **(A)** Presence of cardiovascular disease in RA patients. **(B)** Total atherosclerotic cardiovascular disease in RA patients across 5 geographic regions.

In all patients, any CVD was present in 17% and ASCVD in 15%. The most common ASCVD was CHD and PAD (7–8%), while stroke, heart failure and atrial fibrillation were present in 3–4% (**Figure 2A**). ASCVD showed considerable geographic variation, being very low in India (2%) and Mexico (3%) but nearly 40% in Russia (**Figure 2B**).

### Cardiovascular disease risk factors

CVD risk factor levels are reported in **Table 1**. Mean BMI was 27.4 kg/m^2^. The highest mean BMI was recorded in North America, while the lowest BMI values were found in the Asian cohort (mean 29.7 kg/m^2^ and 23.8 kg/m^2^, respectively). However, the mean BMI in the total cohort was >25 kg/m^2^, which is classified as overweight. In other words, less than 50% were of normal weight and this was stable with increasing age (**Figure 1B**).

Mean TC was 5.0 mmol/L, while mean LDL-c ranged from 2.5 mmol/L in North America to approximately 3.0 mmol/L in Central and Eastern Europe. In the whole cohort, few had hypercholesterolemia (< 1%) with TC>8.1 mmol/L. Although the presence of an LDL-c level > 2.5 mmol/L varied across the world regions (46% in Mexico and 73% in Central and Eastern Europe), after 50 years of age the proportion of patients with LDL-c > 2.5 mmol/L decreased (**Figure 1C**).

BP was on average in the normal range, 128/77 mmHg. Nevertheless, hypertension (BP >140/90 mmHg, and/or self-reported hypertension, and/or use of antihypertensive medication) was present in 2/3 of the patients, this proportion being highest in Central and Eastern Europe (75%) and lowest in Mexico and Asia (36% and 49%, respectively). Overall, BP >140/90 mmHg became more common with age (**Figure 1D**). Of patients not using antihypertensive medication, an average of 17% had BP > 140/90 mmHg, varying from 13% in Mexico to 25% in Central and Eastern Europe.

Current smoking was most common in Central and Eastern Europe (29%), and least common in Asia, Mexico and North America (8–10%). However, North America had a higher proportion of previous smokers compared to the other regions (34%). The number of never smokers was highest in Mexico and Asia (78% and 85%, respectively). There was a decline in current smokers with increasing age (**Figure 1E**).

On average, a high proportion of patients reported moderate or more than moderate physical activity (58%). In Mexico and North America, however, the majority reported less than moderate physical activity (70% and 65% respectively).

The prevalence of diabetes mellitus was on average 13% and comparable across the geographical areas. This comprised mainly type II diabetes (12%). Although the number of patients with HbA1c above recommended levels was low, it increased slightly with increasing age (**Figure 1F**).

**Figure 3.**
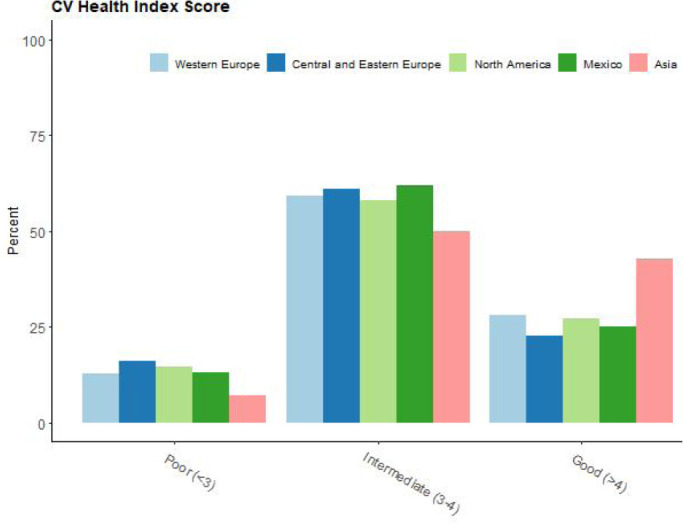
Cardiovascular Health Index Score in RA patients across geographic regions The Cardiovascular Health Index Score (CHIS) was defined by six risk factors at recommended target/levels; blood pressure <140/90 mmHg or <140/80 mmHg for patients with diabetes mellitus, low density lipoprotein cholesterol <2.5 mmol/L, glycated haemoglobin (HbA1c) <7% or glucose <7 mmol/L if HbA1c was not available, non-/ex-smoker, body mass index <25 kg/m^2^, moderate/vigorous physical activity. The number of risk factors at recommended targets/levels was summed and CHIS categories were defined as: poor if <3, intermediate: 3–4, good: 5–6.

The distribution of patients in the different CVD risk categories by SCORE was significantly different across the geographic regions (**Table 1**). Central and Eastern Europe and North America had the worst CVD risk profile according to SCORE (very high and high CVD risk at 58% and 65%, respectively). In comparison, Mexico and Asia had the most favourable CVD risk profile (36% and 29% having very high and high CV risk, respectively). The number of risk factors at target as evaluated by CHIS was also comparable across the geographic regions, regarding poor and intermediate CHIS. Notably, only ¼ had > 4 risk factors at recommended level/target, except in Asia were half of the patients had good CHIS (**Figure 3**).

Patients with ASCVD were on average older and more often male than patients without ASCVD (**Supplementary Table 4**). Evaluating the CVD risk factors in patients with and without ASCVD revealed that patients with ASCVD had somewhat lower lipid levels, more hypertension and diabetes, and were less often physically active compared to those without ASCVD (**Supplementary Table 4**).

## MEDICATION

### Cardiovascular disease preventive medication

Approximately a quarter of all RA patients were on statin therapy, except from the Asian and Mexican cohorts (6% and 16%, respectively). Use of other lipid-lowering agents than statins was infrequent in all continents (1–7%) (**Table 2**).

**Table 2.** Cardiovascular medication across world regions.

	**All**	**Western Europe**	**Central and Eastern Europe**	**Mexico**	**North America (USA and Canada)**	**Asia**	**p-value^a^**
Number of patients in each region	14503	8493	923	407	4030	650	
**Lipid lowering treatment** % [n]							
Any statin	23.9 [10439]	22.2 [4432]	27.0 [921]	15.5 [407]	28.8 [4029]	6.0 [650]	<0.001
Any other lipid lowering agent	2.5 [7831]	2.9 [1833]	1.5 [921]	6.9 [407]	2.3 [4020]	1.2 [650]	<0.001
**Any anti-hypertensive medication** % [n]	29.0 [14503]	17.4 [8493]	57.0 [923]	24.8 [407]	46.8 [4030]	31.8 [650]	<0.001
**Anti-angina medication** % [n]							
Any nitrate	3.1 [7431]	1.4 [1431]	1.6 [922]	0.0 [407]	4.7 [4021]	0.8 [650]	<0.001
**Anti-diabetic medication** % [n]							
Any insulin	3.2 [7831]	2.7 [1832]	2.2 [921]	2.9 [407]	3.7 [4021]	2.9 [650]	0.089
Any oral antidiabetic agent	8.0 [7832]	6.3 [1833]	7.4 [921]	10.8 [407]	8.7 [4021]	7.7 [650]	0.005
**Anti-thrombotic medication** % [n]							
Any anti-platelet	9.3 [7833]	14.1 [1833]	21.6 [921]	1.2 [407]	4.6 [4022]	12.0 [650]	<0.001
Any anti-coagulant	4.7 [7792]	4.0 [1794]	3.3 [921]	0.2 [407]	6.3 [4020]	1.7 [650]	<0.001

a Inter-regional differences.

The use of any antihypertensive treatment also differed substantially between the cohorts, with a mean prevalence of 29%, and lowest use reported in Western Europe and Mexico (**Table 2**).

Use of nitrates for angina pectoris was higher in North America (5%) compared to almost no use in Mexico and Asia.

Use of any antidiabetic medication was comparable across the geographic regions, on average 3% using insulin and 8% using any oral antidiabetic agent, although the latter was somewhat higher in Mexico (11% using an oral antidiabetic agent) (**Table 2**).

### Anti-rheumatic medication

The number of patients receiving methotrexate was high, especially in Central and Eastern Europe and Mexico (73% and 80% respectively) (**Supplementary Table S2**). Treatment with biologic DMARDs was most frequent in Western Europe (42%), but these agents were rarely used in Mexico and Asia (5% and 6%, respectively). The opposite was observed for prednisolone, which was used by 48% in Central and Eastern Europe and 63% in Mexico, while 35–38% used glucocorticoids in Western Europe, North America, and Asia. Usage of non-steroidal anti-inflammatory drugs (NSAIDS) ranged from 15–28% in Western Europe and Mexico but was much more commonly used in Central and Eastern Europe, North America, and Asia (56–63%) (**Supplementary Table S2**).

### Missing data

Missing data on premature CVD, lipids, BP, physical activity, and diabetes was most common in North America and Western Europe, although a lack of HbA1c recording was common in all geographic regions (**Supplementary Table S5**).

## DISCUSSION

CVD is a major comorbidity in patients with RA, and in this large international audit, we describe the state of CVD risk factors and their management in patients with RA across three continents. Our main finding is that CVD risk factors are still highly prevalent in this patient population. Nearly half of the RA patients were categorized into high or very high CVD risk classes according to the SCORE algorithm, and established CVD was present in one sixth of patients. We demonstrated that room for improvement exists for control of CVD risk factors for many, since three out of four patients had poor or intermediate CHIS scores (≤4/6 CVD risk factors at target level). The SURF audit structure proved feasible in patients with RA in the setting of rheumatology and cardiology outpatient clinics, comparably to that in patients with CHD.^9,10^

Cholesterol levels are one of the key CVD risk factors, and should be evaluated among RA patients at a minimum of 5-year intervals and if major changes to antirheumatic treatment occur.^12^ The desired lipid targets in RA are the same as in the general population. Presumably related to systemic inflammatory response, RA patients commonly have low cholesterol levels.^13–15^ As in the general population, some patients with RA have an increased risk of CVD even at low levels of cholesterol.^16^ Nevertheless, in the present study, 63% of RA patients had an LDL-c> 2.5 mmol/L, which is substantially higher than what has been described in other RA patient cohorts,^17^ and more recent surveys from the general population (34–37%).^9,18^ This may suggest that management of hypercholesterolemia among RA patients is inadequate, especially when considering that nearly half of the patients in SURF-RA were categorized high or very high risk classes according to SCORE algorithm.

Tobacco smoking is a major risk factor for CVD.^19^ While smoking prevalence is declining across European countries, 17% of RA patients in this survey are still current smokers. Furthermore, current smoking in Central and Eastern Europe was 1.5 to 3 times more prevalent than in the other regions. Smoking may be of exceptional harm to RA patients: not only are they especially susceptible to CVD events,^20,21^ smoking may also worsen RA-related outcomes.^20,22^

Previous studies have reported that approximately 60% of RA patients are either overweight or obese.^23^ Approximately 25% of patients were characterised as obese (BMI ≥30 kg/m^2^) in our survey, which is lower than reported for patients with CHD in both EUROSPIRE V18 and SURF-CHD,^9^ but comparable to that described for the general population in Europe, where the average age-standardised BMI in 2017 was 25.8–26.8 kg/m^2^.^24^ While being overweight or obese are risk factors for CVD in the general population, both harmful^25^ and cardio-protective^26^ effects of overweight/obesity have been shown in patients with RA.

Diabetes was present in 13% of the SURF-RA cohort, which is in line with that reported in RA patients from one large US-based study,^27^ but twice as high as that reported in another RA cohort from the US.^28^ Diabetes is linked to overweight and obesity, but it may also be a side-effect of glucocorticoid use. Due to the effectiveness of newer anti-rheumatic medications and local joint injections, the use of glucocorticoids in patients with RA is declining, but was still used by nearly 40% in this survey.

In addition to traditional CVD risk factors, inflammation drives the RA-related CVD risk, and effective suppression of disease activity is key to lower CVD risk among RA patients.^12^ Treatment with biologic DMARDs was most frequent in Western Europe and rarely used in Mexico and Asia, which may be related to differences in health care resources. Concordantly, Mexican and Asian RA patients had slightly higher DAS28, ESR and CRP compared to other world regions. The question of whether biologic DMARDs have positive or negative effects on CVD risk in RA is debated.^29–31^ It has been reported that BP is adversely affected by systemic inflammation and there is accumulating evidence suggesting that hypertension is more common in RA patients compared to the general population.^32–35^ Treat-to-target and tight control of RA disease activity has been effectively implemented, as novel anti-rheumatic medications have been developed in the last decades. These innovative RA disease monitoring strategies may explain the quite low overall disease activity levels across the 3 world continents in our survey. The reported prevalence of hypertension in our patients was high (62%) and possibly related to the systemic inflammation in RA and moderately increased CRP levels (mean 2.9 mg/dL). Although this level of CRP has been shown to increase BP^34^ and promote CVD risk in the general population,^36^ it reflects a modest degree of inflammation for RA patients.

In this large international audit, we revealed that the prevalence of ASCVD was particularly high in regions where the presence of CVD risk factors was also high. In Central and Eastern Europe and North America, the presence of ASCVD was 21% and 16%, respectively. Not surprisingly, the percentages of patients having high and very high risk of CVD according to SCORE algorithm were also highest in these two regions, mirroring the high presence of CVD risk factors. Percentages of patients at very high or high CVD risk were 65% in North Americans, 58% in Central and Eastern Europeans, and 39% in Western Europeans, as compared to 29% in Asians and 36% in Mexicans. These differences are explained by Asian and Mexican RA patients being less frequent smokers, having lower BP and TC, and being less obese compared to North American and Central and Eastern European RA patients. Overall, only ¼ had more than 4 CVD risk factors at recommended levels, reflecting inadequate risk factor control.

The limitations of a survey such as SURF-RA should be noted. The variations of the recorded prevalence of CVD risk factors across the 3 continents may reflect the various settings for CVD risk factor recording. For example, the risk factors reported from North America were mostly extracted from primary care patient records, while SURF-RA centres in Western Europe were mostly hospital rheumatology outpatient clinics, and several cardiology outpatient clinics. The centres participating in the audit were either invited through participation of the ATACCRA network (www.atacc-ra.com) or invited through conference contacts. Therefore, the representativeness of the cohorts in relation to nations or geographic regions is not complete. It is desirable that a data sampling frame to be as representative as possible, but since this was a clinical audit without funding, this was not possible and is a limitation to the project. Also, the included number of patients from the various regions varied. Mexico recruited few patients in comparison with the other regions and data may therefore not be representative for RA patients in this region. On the other hand, a strength of the study is that the three countries that recorded the highest number of patients were geographically spread (USA: n=3226, Greece: n=3286, Norway: n=3544). Audits are not epidemiologic studies and lack control groups, and thus a limitation to this audit is the lack of a non-RA control group. Another limitation is the relatively long inclusion period (2014–2019) in which CVD risk and disease prevalence in RA populations could fluctuate, however these data are of interest as data from comparable international cohorts in this time period is lacking. Some of the data were extracted from pre-existing registries, which may explain the differences in missing data across the geographic regions. On another note, the rates of missing data also raise an important point, namely that even in centres with a focus on the CVD aspect of RA disease, these crucial clinical variables are seldom recorded, thus underlining the importance of increasing the awareness of this clinical field. It should be stressed that one of the objectives of an audit, as opposed to an epidemiological study, is to define the degree of missing information, so that improving data collection becomes part of process improvement. The seemingly poor appreciation of CVD risk in RA patients may be due to low knowledge among health personnel,^37^ and due to diffusion of responsibility for CVD risk evaluation between general practitioners, cardiologists and rheumatologists. A clinical audit as ours has limitations in the lack of standardized instruments for all centres such as blood pressure monitors, height and weight measuring scales and no central laboratory measurements. Despite this, clinical audits are recognised as a valuable tool in improving clinical performance in implementing guideline recommended procedures.

To be able to compare data from different regions we decided to use the European definitions of CVD risk calculation, although, SCORE is not validated for use outside Europe. This is a methodological limitation. The strengths of this survey are the large number of patients from various countries, and that the collection is from the last decade, which gives up-to-date information.

## CONCLUSION

Using data collected in the SURF-RA, we have shown that there is still a high prevalence of CVD risk factors and ASCVD in patients with RA across 3 continents, and that there is an unmet need for vigilance and improved implementation of preventive measures in this high CVD risk patient population. Substantial geographical differences were revealed with regard to prevalence of CVD risk factors and established CVD, and the high risk of CVD in RA patients from Central and Eastern Europe is of special concern. We hope that this effort to present the CVD risk factor burden will serve to inform and support a global community of cardiovascular health personnel in the ongoing quest for improved cardiovascular health in patients with RA. Clinical audits are only of value if process improvement and better outcomes result. Therefore, we suggest: (a) the development of Standard Operating Procedures for risk evaluation and management in RA subjects and (b) periodic re-audits to monitor change in risk factors and prevalence of established CVD.

## Figures and Tables

**Supplementary Table S1.** Number of patients by centre, country, and geographic region.

**Geographic region**	**Country**	**Number of Centres**	**Number of Patients**

**Western Europe**	Belgium	2	877
	Greece	17	3286
	Ireland	1	39
	Italy	1	167
	Malta	1	38
	Netherlands	1	180
	Norway	10	3544
	Spain	1	115
	Sweden	1	98
	United Kingdom	1	149

**Central and Eastern Europe**	Czech Republic	4	414
	Russia	3	397
	Slovakia	1	112

**Latin America**	Mexico	2	407

**North America**	Canada	2	236
	USA	2	3794

**Asia**	China	1	454
	India	1	119
	Kyrgyz Republic	1	77

**5**	**19**	**53**	**14503**

**Supplementary Table S2.** Ethnicity and RA disease-related factors across regions.

	**All**	**Western Europe**	**Central and Eastern Europe**	**Mexico**	**North America (USA and Canada)**	**Asia**	p-value*
Number of patients in region	14503	8493	923	407	4030	650	
Ethnicity % [n]							
Arab	0.3 [10879]	0.7 [4897]	0.0 [920]	0.0 [407]	0.1 [4007]	0.0 [648]	
Asian	6.6 [10879]	0.5 [4897]	0.3 [920]	0.0 [407]	1.2 [4007]	100.0 [648]	
Black	0.5 [10879]	0.4 [4897]	0.0 [920]	0.0 [407]	0.9 [4007]	0.0 [648]	
Caucasian	81.9 [10879]	97.3 [4897]	99.7 [920]	0.2 [407]	80.6 [4007]	0.0 [648]	
Hispanic	5.2 [10879]	0.0 [4897]	0.0 [920]	99.8 [407]	14.0 [4007]	0.0 [648]	
Other	5.4 [10879]	1.1 [4897]	0.0 [920]	0.0 [407]	3.2 [4007]	0.0 [648]	
**RA-specific factors**							
Rheumatoid factor [n]	13022	7026	914	407	4028	647	
positive %	56.6	54.1	77.4	87.0	49.0	82.4	<0.001
unknown %	5.5	3.5	2.6	4.7	10.6	0.6	
ACPA [n]	13501	7505	919	407	4026	644	
Positive %	53.9	53.1	65.9	75.2	46.1	81.8	<0.001
unknown %	13.1	14.5	14.6	3.2	11.8	7.8	
Disease duration (years) mean (SD) [n]	10.8 (9.5) [10780]	10.5 (9.5) [8096]	12.1 (9.3) [870]	9.9 (7.5) [406]	12.6 (9.8) [773]	10.5 (9.8) [635]	<0.001
DAS28-CRP mean (SD) [n]	2.6 (1.2) [8122]	2.5 (1.1) [4298]	2.9 (1.2) [824]	2.8 (1.3) [403]	2.8 (1.2) [2485]	2.8 (1.4) [112]	<0.001
DAS28-ESR mean (SD) [n]	3.0 (4.9) [10463]	3.0 (6.1) [6595]	3.3 (1.4) [826]	3.3 (1.6) [309]	2.8 (1.5) [2494]	3.7 (1.3) [239]	<0.001
CRP mg/L median (IQR) [n]	2.9 (1.0–6.2) [9791]	2.1 (1.0–6.0) [4810]	3.6 (1.3–8.0) [866]	0.6 (0.2–1.2) [402]	2.9 (2.9–5.8) [3251]	13.3 (3.2–40.8) [462]	<0.001
ESR mm/Hr median (IQR) [n]	15.0 (7.0–29.0) [12191]	16.0 (7.0–28.0) [6994]	16.0 (8.0–28.0) [872]	18.0 (9.0–31.0) [402]	11.0 (5.0–24.0) [3293]	35.5 (18.0–60.0) [630]	<0.001
**Anti-rheumatic medication** % [n]							
NSAIDS	37.3 [10803]	15.3 [4831]	59.6 [909]	27.5 [407]	55.5 [4018]	63.0 [638]	<0.001
Methotrexate	60.9 [12817]	62.6 [6808]	73.2 [922]	79.9 [407]	54.6 [4030]	52.9 [650]	<0.001
Any other synthetic DMARD	34.8 [12817]	25.1 [6808]	20.5 [922]	41.8 [407]	48.6 [4030]	66.5 [650]	<0.001
**Biologic DMARD** [n]	12333	6577	802	406	3976	572	<0.001
now %	31.0	42.0	36.0	4.9	18.2	5.6	
never %	57.4	52.3	58.9	94.8	57.2	87.9	
Previous %	11.6	5.7	5.1	0.2	24.6	6.5	
**Prednisolone**	12676	6775	899	380	3977	645	<0.001
now %	38.0	35.2	47.7	62.6	38.4	38.1	
never %	2.4	1.7	7.8	3.4	1.3	9.8	
previous %	59.5	63.2	44.5	33.9	60.4	52.1	

**Abbreviations:**RA, rheumatoid arthritis; ACPA, anti-citrullinated protein antibody; DAS28, disease activity score using 28 joints; CRP, C-reactive protein; ESR, erythrocyte sedimentation rate, CVD, cardiovascular disease, [n], number of patients with available data, SD, standard deviation, IQR, inter-quartile range, NSAIDS, non-steroidal anti-inflammatory drugs, DMARD, disease modifying anti-rheumatic drug, [n], number of patients with available data,

* inter-regional differences

**Supplementary Table S3a.** Demographic and RA disease specific data on patients from established cohorts.

	**All**	**West Europe**	**Central and Eastern Europe**	**Latin America**	**North America (USA and Canada)**	**Asia**	**p-value**
**Number of patients**	4528	2584	568	236	843	297	
**Age mean (SD)**	58.5 (13.6)	59.9 (13.8)	59.9 (12.2)	51.1 (12.6)	57.1 (13.4)	52.4 (12.4)	
**[n]**	[4521]	[2578]	[568]	[236]	[843]	[296]	
**Sex [n]**	4514	2576	562	236	843	297	<0.001
**female (%)**	75.4	71.6	79.7	91.9	78.8	77.4	
**male (%)**	24.6	28.4	20.3	8.1	21.2	22.6	
***RA-specific factors***							
**Rheumatoid factor positive [n]**	4066	2136	559	236	841	294	<0.001
**yes (%)**	67.6	61.3	77.8	85.6	66.5	82.3	
**no (%)**	29.5	36.8	21.3	6.8	27.3	16.7	
**unknown (%)**	2.9	1.8	0.9	7.6	6.2	1.0	
**ACPA positive [n]**	4261	2326	565	236	841	293	<0.001
**yes (%)**	66.1	65.2	63.9	78.0	62.8	77.8	
**no (%)**	27.8	31.6	22.3	16.9	30.4	9.6	
**unknown (%)**	6.1	3.2	13.8	5.1	6.8	12.6	
**Disease duration (yrs) mean (SD) [n]**	11.1 (9.6) [4080]	10.9 (9.8) [2542]	12.5 (9.4) [538]	9.7 (6.9) [236]	12.6 (10.4) [480]	8.5 (8.3) [284]	<0.001
**DAS28-CRP mean (SD) [n]**	2.6 (1.2) [3500]	2.5 (1.1) [2021]	2.8 (1.3) [488]	2.5 (1.2) [234]	2.9 (1.3) [645]	2.8 (1.4) [112]	<0.001
**DAS28-ESR mean (SD) [n]**	3.1 (8.4) [3384]	2.9 (11.4) [1825]	3.2 (1.4) [481]	2.9 (1.6) [236]	3.3 (1.4) [603]	3.7 (1.3) [239]	<0.001

**Supplementary Table S3b.** Demographic and RA disease-specific data on consecutively examined patients.

	**All**	**West Europe**	**Central and Eastern Europe**	**Latin America**	**North America (USA and Canada)**	**Asia**	**p-value**
**Number of patients**	9975	5909	355	171	3187	353	
**Age mean (SD) [n]**	60.4 (13.6) [9922]	61.1 (13) [5858]	56.9 (10.9) [355]	55.2 (9.5) [170]	60 (15.1) [3187]	58.5 (13) [352]	<0.001
**Sex [n]**	9901	5836	355	171	3187	352	<0.001
**female (%)**	74.1	75.3	76.6	93.0	70.4	77.3	
**male (%)**	25.9	24.7	23.4	7.0	29.6	22.7	
***RA-specific factors***							
**Rheumatoid factor positive [n]**	8956	4890	355	171	3187	353	<0.001
**yes (%)**	51.6	50.9	76.6	88.9	44.3	82.4	
**no (%)**	41.7	44.8	18.0	10.5	44.0	17.3	
**unknown (%)**	6.7	4.2	5.4	0.6	11.7	0.3	
**ACPA positive [n]**	9240	5179	354	171	3185	351	<0.001
**yes (%)**	48.3	47.6	69.2	71.3	41.7	85.2	
**no (%)**	35.5	32.8	15.0	28.1	45.2	11.1	
**unknown (%)**	16.3	19.6	15.8	0.6	13.1	3.7	
**Disease duration (yrs) mean (SD) [n]**	10.6 (9.4) [6700]	10.3 (9.4) [5554]	11.5 (9) [332]	10.3 (8.2) [170]	12.6 (8.6) [293]	12.2 (10.5) [351]	<0.001
**DAS28-CRP mean (SD) [n]**	2.6 (1.2) [4622]	2.5 (1.0) [2277]	3.0 (1.2) [336]	3.3 (1.2) [169]	2.7 (1.2) [1840]	NaN (NA) [0]	<0.001
**DAS28-ESR mean (SD) [n]**	3 (1.4) [7079]	3.1 (1.4) [4770]	3.5 (1.3) [345]	4.5 (1.2) [73]	2.7 (1.5) [1891]	NaN (NA) [0]	<0.001

**Supplementary Table 4.** Risk factors in patients with and without atherosclerotic cardiovascular disease.

	**With ASCVD**	**Without ASCVD**
Number of patients in region	1681	10944
Age mean(SD) [n]	69 (10.5) [1673]	58.8 (13.6) [10898]
Female sex [n]	60.1	77.3
**Lipids** (mmol/L) median (IQR) [n]		
Total cholesterol	4.5 (3.8–5.3) [1197]	5 (4.3–5.7) [6686]
LDL cholesterol	2.3 (1.8–3.1) [1127]	2.9 (2.3–3.5) [6496]
HDL cholesterol	1.4 (1.1–1.7) [1128]	1.5 (1.2–1.8) [6500]
Triglycerides	1.3 (1–1.8) [1150]	1.2 (0.9–1.7) [6566]
LDL cholesterol >2.5 mmol/L (%) [n]	43.4 [1098]	65.6 [6496]
Total cholesterol >8.1 mmol/L (%) [n]	1.0 [1168]	0.7 [6686]
**Blood pressure** (mmHg) mean (SD)[n]		
Systolic blood pressure	129.9 (18.5) [1439]	127.3 (18.1) [8260]
Diastolic blood pressure	75.2 (12.6) [1422]	77.5 (12.2) [8212]
Blood pressure >140/90 mmHg (%) [n]	33.2 [1426]	29.1 [8230]
Blood pressure >140/90 and no use of anti-hypertensive medication (%) [n]	10.2 [1426]	17.5 [8230]
Hypertensive patients^a^ (%)[n]	88.3 [1578]	59.9 [8951]
**Diabetes** (%) [n]		
Diabetes type I and II combined	25.5 [1483]	11.1 [9299]
Type I diabetes	0.8 [1428]	0.6 [8804]
Type II diabetes	25.1 [1441]	10.3 [9101]
**Glucose** (mmol/L) mean(SD)[n]	6.1 (2) [852]	5.6 (1.8) [3836]
Non-diabetics	5.5 (1) [609]	5.3 (1) [3107]
Diabetics	7.6 (3) [230]	7.9 (3.3) [488]
**HbA1c** (%) mean(SD)[n]	6.2 (1.4) [338]	5.7 (1.4) [1656]
Non-diabetics	5.4 (0.8) [155]	5.2 (0.8) [1206]
Diabetics	6.9 (1.5) [179]	7.1 (1.8) [389]
**Physical activity** [n]	468	3125
less than moderate %	45.7	41.9
moderate %	44.0	40.5
more than moderate %	10.3	17.5
**Smoking** [n]	1551	9964
current %	16.6	17.1
previous %	36.2	23.6
never %	47.3	59.3
**Physical measurements** mean (SD) [n]		
BMI (kg/m^2^)	28.2 (6.4) [1408]	27.6 (5.9) [8385]
Waist circumference (cm)	95.1 (16.5) [305]	91.3 (14.9) [2176]
**Premature CVD in family** % [n]	13.6 [513]	5.3 [3834]

Abbreviations: LDL, low density lipoprotein; HDL, high density lipoprotein; HbA1c, hemoglobin A1c; BMI, body mass index; CVD, cardiovascular disease; SCORE, systematic coronary risk evaluation; CV, cardiovascular; [n], number of patients with available data; SD, standard deviation; IQR, inter-quartile range;

a Hypertensive patients; BP >140/90 mmHg and/or use of a-HT.

**Supplementary Table 5.** Number of patients missing recorded data and as percentage of the total population.

**Variable**	**All**	**Western Europe**	**Central and Eastern Europe**	**Mexico**	**North America (USA and Canada)**	**Asia**	**p-value**
Number of patients	14503	8493	923	407	4030	650	
**CVD categories** [ n] (%)							
Coronary heart disease	324 (2.2)	322 (3.8)	1 (0.1)	0 (0.0)	1 (0.0)	0 (0.0)	<0.001
Stroke	1479 (10.2)	1478 (17.4)	0 (0.0)	0 (0.0)	1 (0.0)	0 (0.0)	<0.001
Peripheral vascular disease	1877 (12.9)	1874 (22.1)	2 (0.2)	0 (0.0)	1 (0.0)	0 (0.0)	<0.001
Atrial fibrillation	10473 (72.2)	7177 (84.5)	0 (0.0)	69 (17.0)	3227 (80.1)	0 (0.0)	<0.001
Heart failure	6818 (47.0)	6810 (80.2)	2 (0.2)	0 (0.0)	6 (0.1)	0 (0.0)	<0.001
Total atherosclerotic CVD	7072 (48.8)	7069 (83.2)	2 (0.2)	0 (0.0)	1 (0.0)	0 (0.0)	<0.001
**Lipids (mmol/L)** [ n] (%)							
Total cholesterol	5144 (35.5)	2411 (28.4)	91 (9.9)	1 (0.2)	2520 (62.5)	121 (18.6)	<0.001
LDL cholesterol	5423 (37.4)	2676 (31.5)	224 (24.3)	1 (0.2)	2385 (59.2)	137 (21.1)	<0.001
HDL cholesterol	5410 (37.3)	2658 (31.3)	234 (25.4)	1 (0.2)	2380 (59.1)	137 (21.1)	<0.001
Triglycerides	5434 (37.5)	2736 (32.2)	195 (21.1)	1 (0.2)	2362 (58.6)	140 (21.5)	<0.001
**Blood pressure (mmHg)** [ n] (%)							
Systolic blood pressure	3647 (25.1)	2842 (33.5)	0 (0.0)	1 (0.2)	803 (19.9)	1 (0.2)	<0.001
Diastolic blood pressure	3712 (25.6)	2906 (34.2)	0 (0.0)	1 (0.2)	803 (19.9)	2 (0.3)	<0.001
**Diabetes – all** [ n] (%)	3347 (23.1)	3075 (36.2)	18 (2.0)	2 (0.5)	239 (5.9)	13 (2.0)	<0.001
Type I diabetes	3897 (26.9)	3578 (42.1)	19 (2.1)	1 (0.2)	286 (7.1)	13 (2.0)	<0.001
Type II diabetes	3587 (24.7)	3565 (42.0)	8 (0.9)	2 (0.5)	7 (0.2)	5 (0.8)	<0.001
**Glucose** (mmol/L) n (%)	9670 (66.7)	7637 (89.9)	65 (7.0)	1 (0.2)	1855 (46.0)	112 (17.2)	<0.001
**HbA1c** n (%)	12249 (84.5)	7612 (89.6)	636 (68.9)	241 (59.2)	3202 (79.5)	558 (85.8)	<0.001
Physical activity [ n] (%)	10559 (72.8)	6724 (79.2)	3 (0.3)	3 (0.7)	3702 (91.9)	127 (19.5)	10559 (72.8)
Smoking [ n] (%)	1331 (9.2)	728 (8.6)	8 (0.9)	0 (0.0)	587 (14.6)	8 (1.2)	1331 (9.2)
**Physical measurements** mean (SD) [ n] (%)							
BMI (kg/m^2^)	2947 (20.3)	1155 (13.6)	69 (7.5)	2 (0.5)	1383 (34.3)	338 (52.0)	<0.001
Waist circumference (cm)	11817 (81.5)	7361 (86.7)	80 (8.7)	147 (36.1)	3796 (94.2)	433 (66.6)	<0.001
**Premature CVD in family** (%)	9784 (67.5)	6556 (77.2)	1 (0.1)	0 (0.0)	3227 (80.1)	0 (0.0)	<0.001

[n]: number of patients missing data; % is patients missing data of total population.

## DATA STATEMENT

No data are available. All data relevant to the study are included in the article or uploaded as supplementary information. The data were pseudonymised by each centre before transferred to the data handling centre at Diakonhjemmet hospital, where it is stored on a central server. The data handling manager is Joe Sexton (joe-sexton0@gmail.com) and the data centre leader is Anne Grete Semb (a-semb@diakonsyk.no).

## Supplementary Data S1.

### DATA PROTECTION AND SECURITY

De-identified data was entered into an electronic data collection system facilitated by the University of Oslo, which was adapted by a statistician (JS). Data already collected were transferred to a template Excel sheet after de-identification and transferred electronically to the data manager site.

Current policy for SURF-RA data held by the data manager site was as follows:

1. Data was periodically removed from the electronic data collection site to ensure that minimal data is held on this system/site at any given time.
2. Research data storage at The Data Coordinating Centre (Diakonhjemmet hospital):
  On a central server, the data network is separated into zones (secure, not secure, limited secure). All data was stored in duplicate on encrypted devices. Security components like firewalls, anti-malware, and spam filters protected the data. Researchers used a protected file area of the Storage Area Network (SAN). Each research project had its own storage area, and only the researchers involved in the project had access to the data. Data security was ensured through use of 1) username/password access to data and programs, 2) the ability to log access, and 3) the use of de-identified/pseudonymised data. Back-up of the server is done regularly through the hospitals Information and Communications Technology (ICT) partner; EVRY, who have access to the research server zone only for backup and operating purposes. The EVRY-employees involved in these operations have signed a declaration of confidentiality, and they are granted access to the systems through an internal acceptance procedure.
